# Analysis of Sensory Attributes and Purchasing Decisions of Plant-Based Beverages of Young Consumers in Poland on a Vegan and Traditional Diet

**DOI:** 10.3390/foods14213672

**Published:** 2025-10-28

**Authors:** Krystyna Szymandera-Buszka, Agata Jankowska, Anna Jędrusek-Golińska, Maciej Jarzębski, Aleksandra Karwik, Jacek Anioła, Marek Wieruszewski, Agnieszka Lasota, Jarosław Pawlicz

**Affiliations:** 1 Department of Gastronomy Science and Functional Foods, Faculty of Food Science and Nutrition, Poznań University of Life Sciences, Wojska Polskiego 31, 60-624 Poznań, Poland; krystyna.szymandera_buszka@up.poznan.pl (K.S.-B.); agata.jankowska@up.poznan.pl (A.J.); anna.jedrusek-golinska@up.poznan.pl (A.J.-G.); okarwik194@gmail.com (A.K.); 2 Department of Physics and Biophysics, Faculty of Food Science and Nutrition, Poznań University of Life Sciences, Wojska Polskiego 38/42, 60-637 Poznań, Poland; 3 Department of Human Nutrition and Dietetics, Faculty of Food Science and Nutrition, Poznań University of Life Sciences, Wojska Polskiego 31, 60-624 Poznań, Poland; jacek.aniola@up.poznan.pl; 4 Department Mechanical Wood Technology, Poznań University of Life Sciences, 60-637 Poznań, Poland; marek.wieruszewski@up.poznan.pl; 5 Department of Jaw Orthopedics, Medical University of Lublin, Chodźki 6, 20-093 Lublin, Poland; 6 Department of Orthopedics and Traumatology, Poznań University of Medical Sciences, 28 Czerwca 1956 135/147, 61-545 Poznań, Poland; jarek.pawlicz@gmail.com

**Keywords:** plant-based beverage, eating behaviours, sensory analysis, purchasing decisions, vegan diet, omnivorous diet

## Abstract

This study aimed to analyse young consumers’ behaviour in Poland toward the consumption of plant-based beverages as milk alternatives. The sensory analysis included oat, buckwheat, cashew, almond, soy, pea, rice, coconut, hazelnut, and macadamia nut beverages and their mixtures. Quantitative analysis of sensory desirability and sensory profiling were employed to evaluate plant-based beverages. The study was conducted among young people (aged 18–35) who declared a vegan or omnivorous diet. It was found that the most frequently consumed beverages included oat and soy beverages, both among vegan and traditional (omnivorous) young groups. A significantly lower frequency of plant-based beverage consumption was confirmed in the group of people with an omnivorous diet. The taste characteristics in plant-based beverages are a key factor in driving sensory desirability among young consumers. Analysis of the influence of respondents’ declared diet and gender revealed no significant differences in the desirability of the taste of the plant-based beverages. The consumers rated the coconut, pea, macadamia, oat, and rice–coconut beverages as the most desirable in terms of taste. The lowest taste desirability was confirmed for the soy drink despite its high reported consumption. The importance of this study focused on the local market development in vegan food, as well as its potential due to further consumer expectations.

## 1. Introduction

The constant expansion of the range and the continuous growth of “plant-based” and “vegan/vegetarian” products, intended to replace traditional dairy products, are responses to the increased demand for these alternatives [1]. Plant-based analogues of milk hold the largest market share [2]. Furthermore, local companies focused more on vegan products due to plant-based product development funds (i.e., from European Union grants, etc.). Moreover, many consumers with various health conditions need to modify their dietary habits. The new vegan development and consumer expectation evaluation is highly demanded in Western European countries such as Poland, where, for several decades, a lot of food has been based on animal meat [3,4].

Consumption of plant-based beverages increased most significantly in Europe between 2018 and 2020. The annual growth rate is estimated to have reached 13.1% between 2023 and 2030 [4]. Poland is also witnessing a rapid increase in the number of new plant-based beverages being introduced to the market, advertised as milk substitutes, and placed on shelves near dairy products [3]. The most frequently cited reasons for choosing plant-based dairy alternatives by consumers include allergies (e.g., to cow’s milk protein) and food intolerances (e.g., of lactose), a declaration of following a plant-based diet, and the perception of these products as health-beneficial alternatives to dairy [1]. This product group also benefits from the interest of people suffering from hypercholesterolemia [5,6]. The research also confirms a correlation between the choice of this product group and declarations of their inclusion in the sustainability category. Due to the reasons mentioned above, the market is focusing on producing innovative plant-based alternatives that meet customer demands [7,8]. However, plant-based beverages provide varying amounts of calories, as well as nutrients such as protein, soluble fibre, and minerals [5,9,10]. These products are also characterised by a different sensory profile, related to the raw materials used in their production [11,12,13,14]. Numerous studies indicate that producers struggle to achieve the appropriate sensory characteristics for this product group [14]. Especially, consumers of animal product analogues very often declare a need for similar attributes in plant-based products [7,15,16,17]. This can pose a serious consumption problem [1,18]. The question arises whether the group of people declaring exclusively plant-based diets should be treated as having different sensory requirements from those declaring consumption of plant-based and animal-based products.

Therefore, this study aimed to analyse young consumers’ behaviour toward sensory attributes and purchasing decisions of plant-based beverages as milk alternatives.

The specific objectives have been to establish the frequency of consumption of plant-based beverages, analyse the factors influencing the purchase of plant-based drinks, and assess the taste, aroma, colour, and overall desirability of plant-based beverages as milk alternatives among young people on a vegan or omnivorous diet.

The research hypotheses were adopted, suggesting a relationship between the type of diet (vegan or omnivorous), gender, and attitudes towards plant-based beverages.

## 2. Materials and Methods

### 2.1. Study Design

In the study, the survey research and sensory analysis were used. Quantitative analysis of sensory desirability and sensory profiling was employed to evaluate plant-based beverages. The study was conducted in Poznań University of Life Sciences (Poland) from May to June 2025. The sensory analysis was performed in an appropriately equipped sensory analysis laboratory [19] at the Department of Gastronomy Science and Functional Foods at the Poznań University of Life Sciences. Studies were performed following the Code of Ethics of the World Medical Association, Consent of the Rector’s Committee for the Ethics of Scientific Research Involving Humans (No. 7/2025).

### 2.2. Material

The beverages (13 samples) included in the sensory study were made of oats, buckwheat, cashews, almonds, soy, pea, rice, coconut, hazelnuts, macadamia, and a mixture of these ingredients (rice–coconut, rice–almond, and oat–soy). The products were purchased from the local market.

### 2.3. Methods

#### 2.3.1. Survey Analysis

##### Experimental Conditions

The survey questionnaire was used in the study (Supplementary Material: S1). The questionnaire contained seven closed-ended, single-choice, and multiple-choice questions. One open-ended question was also used, asking about the ranking of factors determining the choice of a plant-based drink, such as price, taste, packaging, friends’ opinions, and ingredients. The ranking method was used, on a five-point scale, with I place for the most important factor and V place for the least important factor. Closed-ended questions addressed, among other things, diet and the reasons for and the frequency of consuming milk substitutes. For the analysis of the reasons for consuming plant-based beverages, multiple-choice questions were used (subjects could select multiple answers). The consumption frequency was assessed using a range reported by the respondents on an eight-point scale: 8—several times a day; 7—once a day; 6—several times a week; 5—once a week; 4—several times a month; 3—once a month; 2—several times a year; 1—less often/never.

##### Participants

The selection of groups was carried out using a stratified method, taking into account the selection of a sample of Polish society in terms of diet type, gender, and age. The exclusion criteria were age (under 18 and over 35), pregnancy, and lactation. The study group consisted of 292 individuals, including both men (47%) and women (53%), aged 18–35, from various regions of Poland. The respondents followed a vegan diet (48%) or an omnivorous diet (52%).

#### 2.3.2. Sensory Analysis

##### Experimental Conditions

The analysis was conducted between 9 a.m. and 3 p.m. The samples were assessed in three sessions, in the following order: four samples, a 0.5 h break, four samples, a 0.5 h break, and five samples. The samples were coded with three-digit numbers, and the serving order was random (ANALSENS, v.4; Gdańsk, Poland). Samples with a 30–10 g mass were placed in coded, transparent containers (50 mL) covered with lids. Unsweetened black tea (temp. ~40 °C) was used as a taste neutraliser between the samples.

##### Participants

The consumer research was conducted with the same panel (290 participants) as for the survey analysis.

The study group of sensory profiling consists of eight sensory experts.

##### Methods

A sensory desirability test was used in the consumer evaluation.

Consumers evaluated the products’ colour, aroma, taste, and overall desirability. A continuous and unstructured linear scale of 10 cm with the following margin denotations: “undesirable”–“highly desirable” was used for the analysis.

Simultaneously, sensory profiling was conducted using a sample’s quantitative descriptive profile, including the description of attributes (colour and taste) and their intensity values [20] by a trained panel (experts). Thirteen for taste (sweet, fatty, bitter, salty, astringent, metallic, cereal, nutty, legume, watery, milky, hay-like, and coconut). The intensity of each score was determined using a continuous and unstructured linear scale of 10 cm, with appropriate margin descriptions. Uniform margin denotations were applied for descriptors: “undetectable—very intensive”.

The presented study included consumer research (each of 292 consumers assessed each tested sample once) and sensory research, in which a team of eight experts evaluated the intensity of all descriptors in three independent replications.

#### 2.3.3. Data Analysis

Statistical analyses were conducted using Statistica (v.13.1, StatSoft, Tulsa, OK, USA). The effects of drink type and diet were analysed. The results of sensory tests were subjected to an analysis of variance (ANOVA), and then post hoc Tukey’s test was applied at a significance level of *p* < 0.05 to compare the means. Principal component analysis (PCA) was used on the data sets from the sensory profiling of products to assess differences and similarities in sensory profiles based on their aroma, colour, and taste descriptors. PCA was also applied to the colour, aroma, taste, and overall desirability data. To determine the strength of the correlation between the variables, Pearson’s linear correlation coefficients (r) were calculated, with the results delineated as follows: r < 0.200, no linear relationship; 0.200 ≤ r < 0.400, weak linear dependence; 0.400 ≤ r < 0.700, moderate linear dependence; 0.700 ≤ r < 0.900, significant linear dependence; and r ≥ 0.900, very strong linear dependence. The significance level was set at *p* ≤ 0.05.

## 3. Results and Discussion

### 3.1. The Survey Analysis

#### 3.1.1. Factors Influencing Young Consumers’ Behaviour of Consuming Plant-Based Beverages

In the analysis of the reasons for consuming plant-based beverages by young consumers, multiple-choice questions were used: “I am on a vegan diet”, “I am lactose intolerant/allergic to dairy”, “I am on a diet that reduces my intake of saturated fatty acids”, “I don’t like cow’s milk”, and positive sensory attributes.

**Figure 1:**
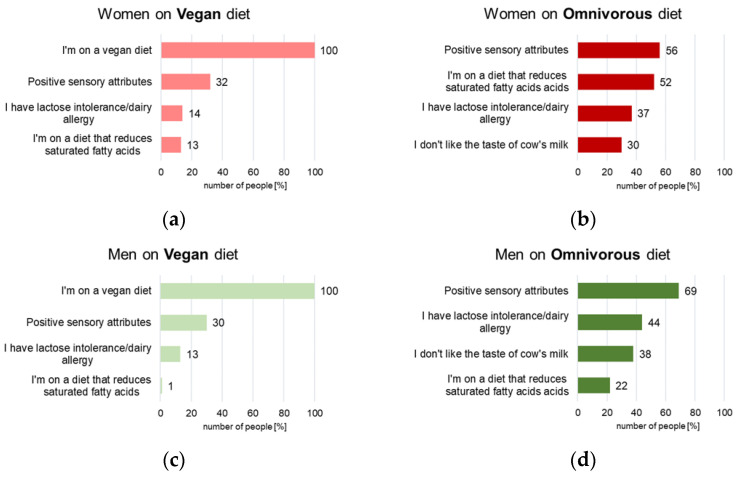
Hierarchy scheme of factors influencing young consumers’ behaviour of consuming plant-based beverages as an alternative to milk [%]: (**a**) women and a vegan diet; (**b**) women and an omnivorous diet; (**c**) men and a vegan diet; (**d**) men and an omnivorous diet (multiple-choice questions).

A statistically significant relationship was confirmed between the declared diet and the analysed factors influencing the choice of plant-based beverages. In contrast, statistical analysis (chi-square test of independence) did not prove a relationship between gender (r = 0.695) and the indicated factors. Based on the obtained research results (Figure 1b), it was found that among women on a diet who declared consumption of both plant and animal products (“omnivorous diet”), the main reasons for consuming plant-based beverages were the positive sensory characteristics of these products (56%) and the use of a diet that lowers saturated fatty acids intake (52%). Research by Madeira et al. confirmed that health benefits and sensory attributes are the primary motivations for consuming plant-based beverages [21]. Thirty-seven percent of women declaring this type of diet indicated lactose intolerance, and 30% of women indicated a dislike of cow’s milk. According to the results obtained from women on a vegan diet (Figure 1a), it was confirmed that 100% of this group attributed their diet as the primary reason for consuming these products. It should be noted that of these individuals, only 32% additionally declared choosing this group of products due to their positive sensory characteristics, 14% due to the indicated lactose intolerance, and 13% use of a diet lowering saturated fatty acids (SFA) intake. However, according to Zaremba et al. [22], 100% of the vegans surveyed also mentioned animal welfare. Other studies also confirm this aspect [23]. Among men with an omnivorous diet, 69% declared their positive sensory characteristics as the main reason for consuming this group of products (Figure 1d). Forty-four percent indicated lactose intolerance, and 38% of men in this group indicated a dislike of cow’s milk, while 22% indicated a diet lowering SFA intake. Analysis of the obtained results confirmed that 100% of the surveyed men who declared a vegan diet indicated that the type of diet was the main reason for consuming this group of products (Figure 1c). Thirty percent of this group confirmed positive sensory characteristics, and 13% confirmed lactose intolerance or a dairy allergy. Only 1% of men with vegan diets confirmed that the main reasons for consuming plant-based beverages were the limitation of saturated fatty acids. Other studies indicate that digestibility problems and lactose intolerance, or lactase enzyme malabsorption, are the primary reasons consumers cite for consuming plant-based milk [6,24,25]. Similar trends were also confirmed among Portuguese respondents aged 18 to 85 [26]. Similar results were obtained regarding the perception of the health impact of plant-based beverages among the Swiss [27].

#### 3.1.2. Analysis of the Most Commonly Consumed Plant-Based Beverages

In the analysis of the frequency of consumption of plant-based beverages as milk alternatives, the following responses were used: several times a day, once a day, several times a week, once a week, several times a month, once a month, several times a year, and less often/never. Based on the results (Table 1), it was confirmed that 21% confirmed that they drank the analysed plant-based beverages less often than several times a month. Another 22% of young respondents declared consuming plant-based beverages once a week, while the lowest percentage declared consuming them several times a day (4%). Taking into account the type of diet reported by respondents, a statistically significant correlation (*p* < 0.005) (Table 1) was confirmed between the declared diet and the frequency of consumption of the assessed milk alternatives. It was confirmed that 22% of those on a vegan diet consumed plant-based beverages at least once a day. However, only 5% of those on an omnivorous diet declared this frequency of consumption.

Firstly, 19.5% of respondents on an omnivorous diet confirmed drinking plant-based beverages less than once a month (Table 1). Among respondents who follow an omnivorous diet, 33% confirmed that they do not consume this group of products (51% of men and 15% of women). Only 6% of people (women) following a vegan diet confirmed consuming this group of products less than once a month. The statistical analysis (chi-square test of independence) did not prove a relationship between gender (r = 0.385) and the frequency of consumption of plant-based beverages.

**Figure 2:**
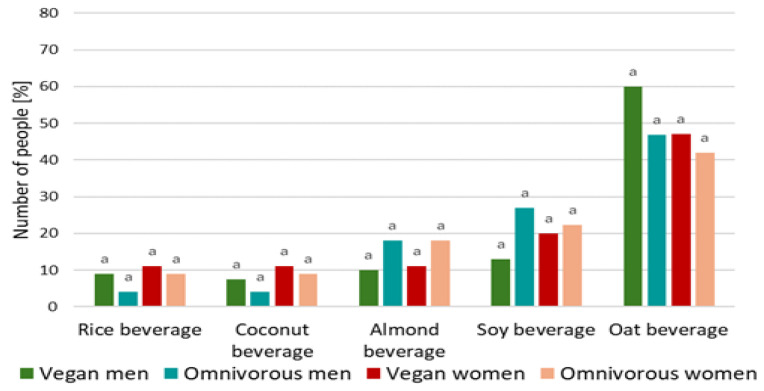
The most commonly consumed plant-based beverages among young consumers with vegan and omnivorous diets; different letters, within the same beverage denote a significant difference at *p* < 0.05 (one-way ANOVA, and post hoc Tukey test).

The results confirmed that the most frequently consumed beverages included almond, soy, rice, coconut, and oat drinks. Based on the results (Figure 2), it was confirmed that respondents most frequently chose oat beverages (49%) and soy beverages (21%).

The lowest percentage of respondents stated that they consumed coconut and rice beverages, at 8% (Figure 2). Statistical analysis (chi-square test of independence) confirmed the lack of a statistically significant relationship between the declared diet (women r = 0.647; men r = 0.019) and gender (vegan diet r = 0.410; omnivorous diet r = 0.334) of the young respondents and the types of milk alternatives they most frequently consumed. It was confirmed that 42% of women with an omnivorous diet declared that they most frequently consume oat beverages (Figure 2). Forty-seven percent of women on a vegan diet also reported oat drink as their most frequently chosen beverage (Figure 2). Among men on a vegan diet, 60% confirmed that they most frequently chose oat drink. Forty-seven percent of men on an omnivorous diet also declared oat drink as their most frequently consumed beverage.

Meanwhile, an analysis of plant-based drink sales in the United States reveals that almond drinks have the highest sales [28]. Soy and almond drinks are also among the most commercialised in Portugal [26]. According to Market insights of The Vegan Society, “The growing plant milk market”, almond and soy beverages each accounted for nearly 40% of the worldwide market value of plant-based drinks in 2019. The oats, rice, hazelnut, coconut, and pea beverages accounted for 20% of the total [29,30].

#### 3.1.3. Analysis of Decision Factors When Choosing a Plant-Based Beverage

In analysing the factors determining the choice of plant-based beverages, a ranking method (five-point scale, I place the most important factor, V place the least important factor) was used for the following responses: price, ingredients, friends’ opinions, sensory qualities, packaging, and others. An open-ended question, “other,” was used to allow for the possibility of identifying other decisive factors when choosing products from this group.

**Figure 3:**
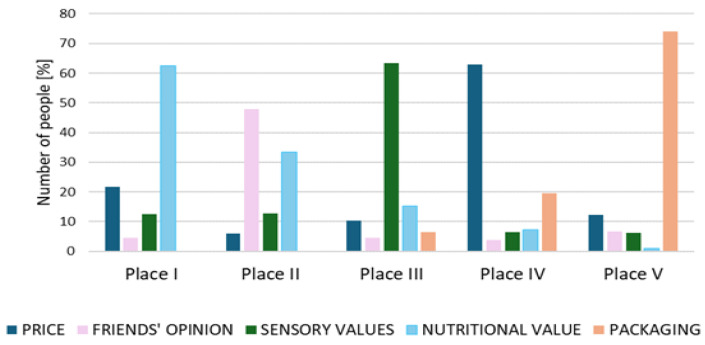
The factors influencing the choice of plant-based beverages among young consumers with vegan and omnivorous diets (five-point scale, I place the most important factor, V place the least important factor).

Based on the results obtained across the entire sample (regardless of diet and gender), it was confirmed that 37% of respondents prioritised the sensory qualities of the beverages they purchased (Figure 3). The largest group (30%) ranked nutrition value as the most crucial factor, and a group of 29% ranked nutritional value third.

Previous studies have also shown that nutritional value is an effective factor in choosing these products [31]. Other studies also indicated that the majority of participants confirmed that these products are a suitable alternative to cow’s milk in terms of sustainability and nutritional value. However, it is noteworthy that a significant portion of this group lacked knowledge about these ingredients and responded, “I have no idea what nutrients are in them”! A significant portion of young consumers also did not pay attention to the nutritional content of the drink [32]. Price was cited as the fourth-most important factor by 31% of respondents. A group of 29% ranked price second in importance, and 14% ranked it first. Packaging-related features were the least important for 66% of respondents. The statistical analysis confirmed a statistically significant effect of diet, as well as gender (only omnivorous diet) (*p* < 0.05), on price as a decisive factor in product selection (Table 2). This relationship was not confirmed for the vegan group (*p* = 0.912). It was confirmed that 40% of women and 31% of men on an omnivorous diet indicated price as the most crucial factor when choosing a plant-based beverage. An additional 28% of women and 58% of men on an omnivorous diet identified this factor as the second most crucial. However, among women and men on a vegan diet, the largest groups (42% and 41%, respectively) indicated this factor as the fourth-most important. The statistical analysis confirmed a statistically significant effect of diet, as well as gender (*p* < 0.05), on the opinion of friends as a decisive factor in product selection (Table 2). Other studies have also confirmed a relationship between the price of these products and purchase frequency, as well as a willingness to choose cheaper products from this range [33,34,35]. Because the production costs of plant-based drinks are higher, the price of plant-based milk substitutes is higher than the price of cow’s milk. Previous studies conducted among the Polish community have confirmed consumer dissatisfaction with the high prices [22].

A group of 35% of respondents indicated the opinion of friends as the second-most crucial factor when choosing plant-based beverages. A statistically significant influence of diet and gender was confirmed as a decisive factor in this product selection. It was found that 60% of women and 36% of men on an omnivorous diet considered the opinion of friends as the second-most crucial factor. Among those on a vegan diet, 40% of women and 35% of men identified this factor as the fourth choice. A statistically significant influence of gender and diet (but only on men’s) (*p* < 0.05) choice of packaging was also confirmed as a decisive factor in product selection. However, none of the respondents indicated packaging as the most crucial factor in product selection. Sixty-four percent of men on a vegan diet and 78% of men on an omnivorous diet indicated packaging as the least important factor when choosing plant-based beverages. The results of the statistical analysis confirmed the statistically significant influence (*p* < 0.05) of diet and gender on sensory qualities as a decisive factor in product selection. It was found that 48% of surveyed women and 41% of men on a vegan diet indicated sensory attributes as the third most crucial factor. In the group on an omnivorous diet, 55% of women and 67% of men stated the sensory qualities of plant-based beverages as the most crucial factor in their choice.

### 3.2. Sensory Analysis

#### 3.2.1. Sensory Consumer Results

**Figure 4:**
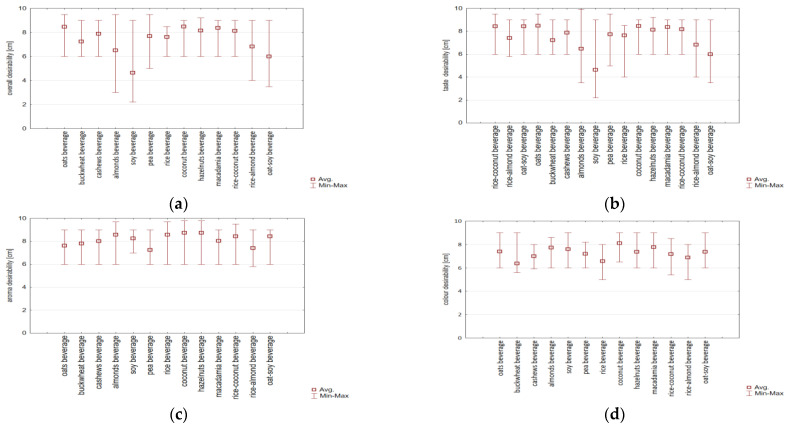
Box plot diagram of consumer desirability of (**a**) overall desirability; (**b**) taste; (**c**) aroma; (**d**) colour of plant-based beverages as an alternative to milk among young consumers with vegan and omnivorous diets.

Consumer analysis was conducted for beverages of oats, buckwheat, cashews, almonds, soy, peas, rice, coconut, hazelnuts, macadamia, and a mixture of these ingredients (rice–coconut, rice–almond, and oat–soy). The study considered analysing the studied products’ colour, aroma, taste, and overall desirability. Based on the research results, the overall desirability was confirmed to be 5.60–8.47 points (Figure 4a; Table S2). Based on the statistical analysis results (one-way ANOVA and post hoc Tukey test), no statistically significant differences (*p* < 0.05) were found in the sensory desirability ratings for all plant-based beverages analysed. The trend was confirmed, however, that the highest desirability was found for the coconut, pea, macadamia, and oat beverages (mean 8.42 points on a 10-point scale). The studies of Pointke et al. [36] also confirm a higher sensory acceptance of oat beverages than some brands of cow’s milk. The high sensory appeal of the macadamia beverage has been confirmed by previous studies [37,38]. Previous studies among people declaring omnivorous diets confirmed the perception of plant-based drinks as “feminine”, and oat milk was described as “boring” and “irritating” in comparison to cow’s milk [15,17]. The lowest desirability was found for the soy beverage, with a mean of 5.60 points (Table S1). However, these differences were not statistically significant. The most considerable variability in ratings was confirmed for the soy (2.2–9.0 points) and oat–soy (3.5–9.0 points), almond (3.0–9.5 points), and rice–almond (4.0–9.0 points) beverages. Statistical analysis (Table 3) confirmed the highest correlation coefficient between overall desirability and taste desirability (r = 0.964 points). This was confirmed for the vegan and omnivore diets. For products that had a higher taste desirability, high overall desirability was confirmed. Based on the research results, the desirability of the taste was confirmed to be 5.63–8.50 points (Figure 4b, Table S2). Similarly to overall desirability, the most considerable variability in ratings of taste desirability was observed for the soy (2.2–9.0 points) and rice–almond (3.5–9.0 points) beverages. Additionally, significant variability of taste ratings was confirmed for the almond (3.5–9.9 points) and rice–almond (4.0–9.0) beverages. Analysis of the influence of respondents’ gender and declared diet revealed no significant differences in the desirability of the taste of the plant-based beverages studied as alternatives to dairy beverages (*p* < 0.05). Both women and men on a vegan diet rated the coconut, pea, macadamia, oat, and rice–coconut beverages as the most desirable in terms of taste (Figure 4c).

The attribute examined was also the aroma of plant-based beverages (Table S2). The results of the sensory aroma desirability assessment across the entire survey group, regardless of gender and dietary preferences, are presented in Figure 4c. Statistical analysis (Table 3) confirmed a low correlation coefficient between overall desirability and aroma desirability (r = 0.105). This was confirmed for the vegan and omnivore diets. Based on the obtained results, no statistically significant differences (*p* < 0.05) were found in the sensory aroma desirability assessment for all analysed plant-based milk analogue beverages. The results confirmed high aroma desirability (5.8–8.5 points on a 10-point scale) for all beverages assessed. A low variability in ratings was confirmed for the aroma of beverages (3.0–3.8 points). Statistical analysis (one-way ANOVA and post hoc Tukey test) of the relationship between gender and reported diet revealed no statistically significant differences in the desirability of the aroma of the plant-based beverages studied. Both men on an omnivorous diet and women on a vegan diet rated all the analysed beverages similarly.

The attribute examined was also the colour of plant-based beverages (Figure 4d, Table S2). Statistical analysis (Table 3) confirmed the lowest correlation coefficient between overall desirability and colour desirability (r = 0.007). This was confirmed for the vegan and omnivore diets. We did not confirm (Figure 4d) significant differences (*p* < 0.05) for the sensory colour desirability assessment for all analysed plant-based milk analogue beverages. The results confirmed high colour desirability (6.38–8.1 points on a 10-point scale) for all beverages assessed. The only trend confirmed was that the coconut beverage had the highest colour desirability (average 8.1 points on a 10-point scale). The rice beverage had the lowest colour desirability, with an average of 6.58 points. Statistical analysis (one-way ANOVA and post hoc Tukey test) of the relationship between gender and reported diet revealed no statistically significant differences in the desirability of the colour of the plant-based beverages studied. Both men on an omnivorous diet and women on a vegan diet rated all the analysed beverages similarly.

#### 3.2.2. Sensory Profiling Results

**Figure 5:**
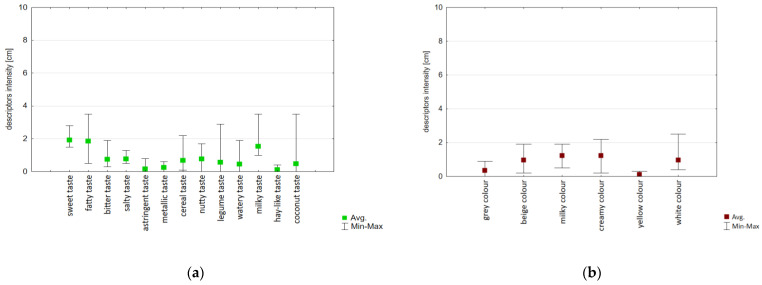
Box plot diagram of sensory profiles of (**a**) taste; (**b**) colour of plant-based beverages as an alternative to milk.

The sensory profiling defined and determined the perception of colour and taste descriptors (Figure 5a,b, Table S2). The analysis confirmed six descriptors for colour (grey, beige, milky, creamy, yellow, and white), 13 for taste (sweet, fatty, bitter, salty, astringent, metallic, cereal, nutty, legume, watery, milky, hay-like, and coconut).

The colour and taste profile assessment confirmed the variability of the identified descriptors and their intensity across the product group. This variability was related to the type of raw material used in the production of the beverage. The greatest range between results was observed for coconut, soy, nut, and grain taste. A similar range between scores was confirmed for white and beige colours.

#### 3.2.3. Relationships Between Descriptive and Consumer Desirability Data

The consumer sensory analysis confirmed that omnivores and vegans have similar attitudes towards the sensory attributes of the investigated plant-based beverages. Statistical analysis confirmed no correlation between overall desirability and the desirability of colour and aroma of these products. However, a high correlation coefficient was found between overall desirability and taste desirability.

**Figure 6:**
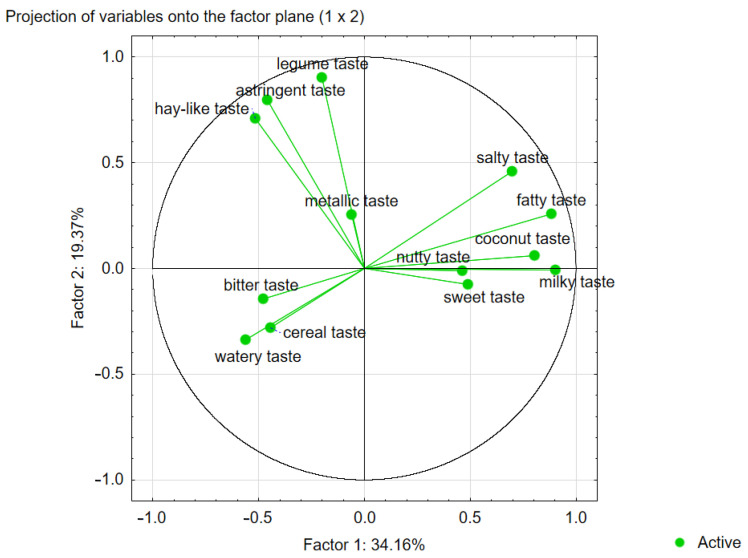
Principal component analysis (PCA) of the loadings plot of the sensory taste descriptors and overall desirability of plant-based beverages as an alternative to milk into factors (F1 × F2).

**Figure 7:**
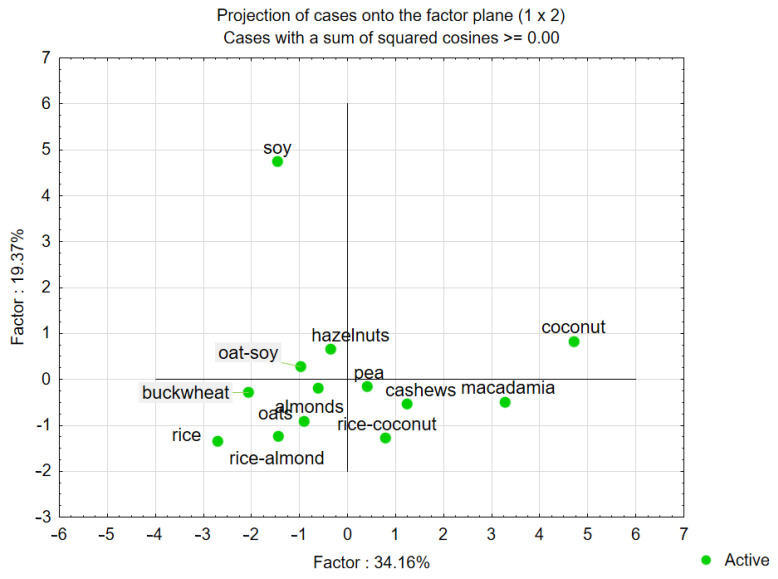
Map of the variants of plant-based beverages as an alternative to milk into factors (F1 × F2). Case–factor coordinate plots based on the attributes of taste profiles (PCA analysis).

Therefore, principal component analysis (PCA) was used to study the relationships between the taste attributes and the characteristic sensory taste profiles of plant-based beverages (variables), and to derive factors according to which these variables can be classified (Figure 6 and Figure 7).

PCA was also used to map the variants tested in our experiment (i.e., samples with analysed plant-based beverages) into these factors. The PCA confirmed that the first two factors (F1 and F2) were the most important elements explaining variation in the data. They explained approximately 52% of the total variance for taste. Therefore, they were selected for data interpretation. The absolute values of factor coordinates for variables indicate the relationship between the factors and the sensory attributes of the analysed beverages (Table 4). For the taste attributes of analysed products, F1 was most strongly related to the taste of milky (r = 0.902), fatty (r = 0.881), coconut (r = 0.812), salty (r = 0.697), and F2 to the taste of legume (r = 0.928), astringent (r = 0.791), and hay-like (r = 0.710).

The analysis confirms the relationship between the plant-based beverage variant and the type and intensity of the defined descriptors. For taste descriptors, the projection of the plant-based beverage variants on the factor plane F1xF2 (Figure 5) confirms that the samples are plotted to the right side of the F1 axis (i.e., they have positive coordinate values for F1). Samples of macadamia beverage were characterised by the highest milky taste intensity (3.5 points), higher intensity of fatty taste (3.2 points), and sweet taste (2.0 points). The samples of coconut beverage were characterised by the highest intensity of coconut taste (3.5 points) and fatty taste (3.5 points), as well as a higher intensity of milky taste (3.2 points) and sweet taste (2.1 points). Moreover, these samples were characterised by the lowest intensity of legume taste (0.2–0.3 points).

The lowest taste desirability was confirmed for the samples of soy beverage. It was confirmed that the samples are plotted to the left side of the F1 axis (i.e., they have negative coordinate values for F1). These samples were characterised by the highest intensity of legume taste (2.9 points), lower milky taste (1.9 points), and fatty taste (1.2 points). A consumer declares a lack of acceptance of the taste characteristic of the raw material from which the drink is made. Previous studies also confirm that negative experiences and unfavourable comparisons with animal-based products can lower expectations for these plant-based analogues [15]. Other studies have also confirmed that the sensory properties of plant-based foods and beverages are among the most important factors in designing this product group [39]. Previous studies also indicate unfavourable taste descriptors perceived by consumers. These results confirm that soymilk can have a “beany” and “painterly” aftertaste resulting from lipoxygenase activity [40]. Aschemann-Witzel et al. [41] also confirmed that products with a higher intensity of nutty taste have lower sensory desirability. For example, studies conducted among consumers who declared a need to reduce lactose consumption confirmed lower sensory acceptability for soy milk compared to lactose-free cow’s milk [31]. The study by Andrés et al. [42] confirmed a decrease in sweet taste intensity with increasing soy content. Other studies have also confirmed a positive correlation between positive attitudes toward plant-based beverages and the intensity of sweet taste, as well as a negative correlation with the tastes of fermented, bitter, and sour [14]. The undesirable taste of soy beverage results from n-hexanol and n-hexanal, which are formed by the oxidation of plant lipids. This descriptor is also associated with the presence of isoflavonoids [7,43]. Yao’s [44] study confirms that young consumers typically do not drink plant-based beverages at home without the addition of plant-based milk for meal preparation. They prefer ready-to-drink beverages based on modified milk, which contain sugar or sweeteners, as well as flavourings. Therefore, they like sweet plant-based milks.

A strength of this study was defining the positive and negative attributes of plant-based beverages, with the highest impact on sensory desirability among consumers, regardless of whether they follow an omnivorous or vegan diet. Another strength was the sample size of sensory consumers, comprising 292 participants, and the analysis, which utilised both survey and sensory data within the same group.

The main limitation was that it did not consider the education of the respondents, which may influence their behaviour toward beverages. Therefore, further research should focus on increasing the variability of groups due to education.

## 4. Conclusions

It was confirmed that a relationship exists between the declared diet and the frequency of consumption of plant-based beverages as milk alternatives. A significantly lower frequency of plant-based beverage consumption was confirmed in the group of people with an omnivorous diet. However, no relationship was found between gender and the frequency of consumption of these beverages.

The most frequently consumed beverages included almond, soy, rice, coconut, and oat drinks.

The surveyed young consumers had similar sensory desires for plant-based beverages, regardless of their diet and gender. Taste was the sensory attribute that most determined the level of sensory desirability of plant-based beverages in both groups.

The young consumers were found to have a positive attitude towards these products due to their “milky” taste, which correlated with a slightly fatty and salty taste. Therefore, an interesting raw material, highly desired by young consumers from a sensory perspective, was the coconut and macadamia beverage. However, this requires more detailed research. The high range in desirability ratings for the almond beverage suggests that entrepreneurs should carefully select their target audience.

## Figures and Tables

**Table 1:** The frequency of consumption of plant-based beverages as milk alternatives among young consumers with vegan and omnivorous diets (n = 292).

	Men		Women		Total Group
	Vegan	Omnivorous	Vegan	Omnivorous	
	Number of Participants [%],				
several times a day	4	0	12	1	4
once a day	14	3	13	6	9
several times a week	17	11	18	19	16
once a week	27	5	39	18	22
several times a month	32	7	5	0	11
once a month	6	4	7	21	10
several times a year	0	19	6	20	11
less often/never	0	51	0	15	17
Pearson’s chi-squared test	diet		*p* < 0.0001		
	gender		*p* = 0.385		

**Table 2:** The Pearson’s chi-squared test values for analysis of decision factors when choosing a plant-based beverage among young consumers with vegan and omnivorous diets.

Comparable Indicators		*p*-Value	Results of Analysis
PRICE			
diet	Women	*p* < 0.05	impact of diet
	Men	*p* < 0.05	impact of diet
gender	Vegan diet	*p* = 0.912	no impact of diet
	Omnivorous diet	*p* < 0.05	impact of gender
FRIENDS’ OPINION			
diet	Women	*p* < 0.05	impact of diet
	Men	*p* < 0.05	impact of diet
gender	Vegan diet	*p* < 0.05	impact of gender
	Omnivorous diet	*p* < 0.05	impact of gender
SENSORY VALUES			
diet	Women	*p* < 0.05	impact of diet
	Men	*p* < 0.05	impact of diet
gender	Vegan diet	*p* < 0.05	impact of gender
	Omnivorous diet	*p* < 0.05	impact of gender
NUTRITIONAL VALUE			
diet	Women	*p* < 0.05	impact of diet
	Men	*p* < 0.05	impact of diet
gender	Vegan diet	*p* < 0.05	impact of gender
	Omnivorous diet	*p* < 0.05	impact of gender
PACKAGING			
diet	Women	*p* = 0.117	no impact of diet
	Men	*p* < 0.05	impact of diet
gender	Vegan diet	*p* < 0.05	impact of gender
	Omnivorous diet	*p* < 0.05	impact of gender

**Table 3:** Correlation coefficients between the overall desirability and colour, aroma, and taste desirability to plant-based beverages as an alternative to milk among young consumers with vegan and omnivorous diets.

Sensory Attributes	Consumer Desirability			
	Vegan		Omnivore	
	Women	Men	Women	Men
taste	0.964	0.996	0.997	0.997
aroma	0.105	0.083	0.040	0.213
colour	0.007	−0.007	−0.024	0.040

**Table 4:** The factor loadings for taste attributes of plant-based beverages as an alternative to milk.

Sensory Attributes	Taste Desirability		Overall Desirability	
	F1	F2	F1	F2
Taste				
sweet taste	0.490	–0.076	0.491	–0.075
fatty taste	0.881 *	0.230	0.883 *	0.258
bitter taste	–0.480	–0.145	–0.480	–0.145
salty taste	0.697	0.460	0.697	0.460
astringent taste	–0.459	0.791 *	–0.458	0.795 *
metallic taste	–0.061	0.256	–0.061	0.256
cereal taste	–0.444	–0.279	–0.444	–0.279
nutty taste	0.462	–0.012	0.462	–0.012
legume taste	–0.202	0.928 *	–0.202	0.903 *
watery taste	–0.562	–0.338	–0.562	–0.338
milky taste	0.902 *	–0.009	0.902 *	–0.009
hay-like taste	–0.515	0.710	–0.516	0.710
coconut taste	0.812 *	0.058	0.803 *	0.059

* are believed to be most important.

## Data Availability

The original contributions presented in this study are included in the article/Supplementary Materials. Further inquiries can be directed to the corresponding author.

## Supplementary Materials

The following supporting information can be downloaded at: https://www.mdpi.com/article/10.3390/foods14213672/s1, Table S1. Mean scores (n = 292) of sensory consumer desirability of colour, aroma, and taste of plant-based beverages as milk alternatives among young consumers with vegan and omnivorous diets. Table S2. Mean scores (n = 8) of sensory taste profiling of plant-based beverages as milk alternatives. S1. Survey questionnaire template.

## References

[1] Geburt K., Albrecht E.H., Pointke M., Pawelzik E., Gerken M., Traulsen I. (2022). A Comparative Analysis of Plant-Based Milk Alternatives Part 1: Composition, Sensory, and Nutritional Value. Sustainability.

[2] Vaikma H., Kaleda A., Rosend J., Rosenvald S. (2021). Market Mapping of Plant-Based Milk Alternatives by Using Sensory (RATA) and GC Analysis. Future Foods.

[3] Adamczyk D., Jaworska D., Affeltowicz D., Maison D. (2022). Plant-Based Dairy Alternatives: Consumers’ Perceptions, Motivations, and Barriers—Results from a Qualitative Study in Poland, Germany, and France. Nutrients.

[4] Sneha Mali|Plant Based Beverage Market Report 2025 (Global Edition). https://www.cognitivemarketresearch.com/plant-based-beverage-market-report#author_details.

[5] Sethi S., Tyagi S.K., Anurag R.K. (2016). Plant-Based Milk Alternatives an Emerging Segment of Functional Beverages: A Review. J. Food Sci. Technol..

[6] Vanga S.K., Raghavan V. (2018). How Well Do Plant Based Alternatives Fare Nutritionally Compared to Cow’s Milk?. J. Food Sci. Technol..

[7] Tangyu M., Muller J., Bolten C.J., Wittmann C. (2019). Fermentation of Plant-Based Milk Alternatives for Improved Flavour and Nutritional Value. Appl. Microbiol. Biotechnol..

[8] Acquah J.B., Amissah J.G.N., Affrifah N.S., Wooster T.J., Danquah A.O. (2023). Consumer Perceptions of Plant Based Beverages: The Ghanaian Consumer’s Perspective. Future Foods.

[9] Smyth P.P.A. (2021). Iodine, Seaweed, and the Thyroid. Eur. Thyroid. J..

[10] Walther B., Guggisberg D., Badertscher R., Egger L., Portmann R., Dubois S., Haldimann M., Kopf-Bolanz K., Rhyn P., Zoller O. (2022). Comparison of Nutritional Composition between Plant-Based Drinks and Cow’s Milk. Front. Nutr..

[11] Bucher T., Müller B., Siegrist M. (2015). What Is Healthy Food? Objective Nutrient Profile Scores and Subjective Lay Evaluations in Comparison. Appetite.

[12] Geburt K., Albrecht E.H., Pointke M., Pawelzik E., Gerken M., Traulsen I. (2022). A Comparative Analysis of Plant-Based Milk Alternatives Part 2: Environmental Impacts. Sustainability.

[13] Appiani M., Cattaneo C., Laureati M. (2023). Sensory Properties and Consumer Acceptance of Plant-Based Meat, Dairy, Fish and Eggs Analogs: A Systematic Review. Front. Sustain. Food Syst..

[14] Pramudya R.C., Lee J., Chapko M.J., Lee K.R., Lee S., Lee J.Y., Tokar T., Seo H.S. (2019). Variations in U.S. Consumers’ Acceptability of Commercially-Available Rice-Based Milk Alternatives with Respect to Sensory Attributes and Food Neophobia Traits. J. Sens. Stud..

[15] Jaeger S.R., Giacalone D. (2021). Barriers to Consumption of Plant-Based Beverages: A Comparison of Product Users and Non-Users on Emotional, Conceptual, Situational, Conative and Psychographic Variables. Food Res. Int..

[16] Giacalone D., Clausen M.P., Jaeger S.R. (2022). Understanding Barriers to Consumption of Plant-Based Foods and Beverages: Insights from Sensory and Consumer Science. Curr. Opin. Food Sci..

[17] Cardello A.V., Llobell F., Giacalone D., Roigard C.M., Jaeger S.R. (2022). Plant-Based Alternatives vs Dairy Milk: Consumer Segments and Their Sensory, Emotional, Cognitive and Situational Use Responses to Tasted Products. Food Qual. Prefer..

[18] Pritulska N., Motuzka I., Koshelnyk A., Motuzka O., Yashchenko L., Jarossová M., Krnáčová P., Wyka J., Malczyk E., Habánová M. (2021). Consumer Preferences on the Market of Plant-Based Milk Analogues. Potravin. Slovak J. Food Sci..

[19] (2010). Sensory Analysis. General Guidance for the Design Studio of Sensory Analysis.

[20] (2016). Sensory Analysis—Methodology—General Guidance for Establishing a Sensory Profile.

[21] Madeira D.S.S., Sousa B.H.G.d., Abreu V.K.G., Lemos T.d.O., Reis A.S., Maciel M.C.G., Dutra R.P., Pereira A.L.F. (2025). Production and Stability of Plant-Based Fermented Beverages Using Babassu Coconut (*Attalea speciosa*) and Cashew Apple (*Anacardium occidentale*) Sweetened with Various Polyols. J. Food Compos. Anal..

[22] Zaremba A., Jędrusek-Golińska A., Kobus-Cisowska J., Szymandera-Buszka K. (2023). Consumer Behavior towards Plant-Based Drinks as Milk Alternatives. Agric. Advis..

[23] Boaitey A., Minegishi K. (2020). Determinants of Household Choice of Dairy and Plant-Based Milk Alternatives: Evidence from a Field Survey. J. Food Prod. Mark..

[24] Rai S.R., Pachisia J., Singh S. (2018). A Study on the Acceptability of Plant-Based Milk and Curd among the Lactose Intolerant People Residing in Kolkata. Int. J. Health Sci. Res..

[25] Haas R., Schnepps A., Pichler A., Meixner O. (2019). Cow Milk versus Plant-Based Milk Substitutes: A Comparison of Product Image and Motivational Structure of Consumption. Sustainability.

[26] Anjos O., Pires P.C.P., Gonçalves J., Estevinho L.M., Mendonça A.G., Guiné R.P.F. (2024). Plant-Based Beverages: Consumption Habits, Perception and Knowledge on a Sample of Portuguese Citizens. Foods.

[27] Ammann J., Grande A., Inderbitzin J., Guggenbühl B. (2023). Understanding Swiss Consumption of Plant-Based Alternatives to Dairy Products. Food Qual. Prefer..

[28] Ramsing R., Santo R., Kim B.F., Altema-Johnson D., Wooden A., Chang K.B., Semba R.D., Love D.C. (2023). Dairy and Plant-Based Milks: Implications for Nutrition and Planetary Health. Curr. Environ. Health Rep..

[29] Sharma N., Yeasmen N., Dubé L., Orsat V. (2024). A Review on Current Scenario and Key Challenges of Plant-Based Functional Beverages. Food Biosci..

[30] Aydar E.F., Tutuncu S., Ozcelik B. (2020). Plant-Based Milk Substitutes: Bioactive Compounds, Conventional and Novel Processes, Bioavailability Studies, and Health Effects. J. Funct. Foods.

[31] Baş M., Kahriman M., Ayakdas G., Hajhamidiasl L., Koseoglu S.K. (2024). Driving Factors Influencing the Decision to Purchase Plant-Based Beverages: A Sample from Türkiye. Foods.

[32] Cummins E., Lane K. (2023). Consumer Knowledge and Perceptions of the Nutrition Content, Sustainability and Price of Non- Dairy, Plant-Based Milk Products: A Mixed-Methods Approach. Proc. Nutr. Soc..

[33] Mohd Azman N.M., Muhammad R., Ramli N., Abu Bakar S.K. (2023). A Review of Product Knowledge and Determinants of Consumer Purchase Intention on Plant-Based Meat Products in Malaysia. Environ. -Behav. Proc. J..

[34] Pope D.H., Karlsson J.O., Baker P., McCoy D. (2021). Examining the Environmental Impacts of the Dairy and Baby Food Industries: Are First-Food Systems a Crucial Missing Part of the Healthy and Sustainable Food Systems Agenda Now Underway?. Int. J. Env. Res. Public Health.

[35] Moss R., Barker S., Falkeisen A., Gorman M., Knowles S., McSweeney M.B. (2022). An Investigation into Consumer Perception and Attitudes towards Plant-Based Alternatives to Milk. Food Res. Int..

[36] Pointke M., Ohlau M., Risius A., Pawelzik E. (2022). Plant-Based Only: Investigating Consumers’ Sensory Perception, Motivation, and Knowledge of Different Plant-Based Alternative Products on the Market. Foods.

[37] Camacho-Teodocio J.D., Gallardo-Velázquez T., Osorio-Revilla G., Castañeda-Pérez E., Velázquez-Contreras C., Cornejo-Mazón M., Hernández-Martínez D.M. (2024). Macadamia (*Macadamia integrifolia*) Nut-Based Beverage: Physicochemical Stability and Nutritional and Antioxidant Properties. Beverages.

[38] Lamas D.L., Álvarez S.T. (2023). Development of Snacks Based on *Macadamia integrifolia* Nuts Enriched with Omega-3. Food Chem. Adv..

[39] Piqueras-Fiszman B., Spence C. (2015). Sensory Expectations Based on Product-Extrinsic Food Cues: An Interdisciplinary Review of the Empirical Evidence and Theoretical Accounts. Food Qual. Prefer..

[40] Mäkinen O.E., Wanhalinna V., Zannini E., Arendt E.K. (2016). Foods for Special Dietary Needs: Non-Dairy Plant-Based Milk Substitutes and Fermented Dairy-Type Products. Crit. Rev. Food Sci. Nutr..

[41] Aschemann-Witzel J., Gantriis R.F., Fraga P., Perez-Cueto F.J.A. (2020). Plant-Based Food and Protein Trend from a Business Perspective: Markets, Consumers, and the Challenges and Opportunities in the Future. Crit. Rev. Food Sci. Nutr..

[42] Andrés V., Tenorio M.D., Villanueva M.J. (2015). Sensory Profile, Soluble Sugars, Organic Acids, and Mineral Content in Milk- and Soy-Juice Based Beverages. Food Chem..

[43] Irondi E.A., Aina H.T., Imam Y.T., Bankole A.O., Anyiam A.F., Elemosho A.O., Kareem B., Adewumi T.O. (2025). Plant-Based Milk Substitutes: Sources, Production, and Nutritional, Nutraceutical and Sensory Qualities. Front. Food Sci. Technol..

[44] Yao Y., He W., Cai X., Bekhit A.E.D.A., Xu B. (2022). Sensory, Physicochemical and Rheological Properties of Plant-Based Milk Alternatives Made from Soybean, Peanut, Adlay, Adzuki Bean, Oat and Buckwheat. Int. J. Food Sci. Technol..

