# Supplementary material for: Analysis of Sensory Attributes and Purchasing Decisions of Plant-Based Beverages of Young Consumers in Poland on a Vegan and Traditional Diet
Source: Foods. 2025 Oct 28;14(21):3672. doi: 10.3390/foods14213672 (PMC12607725; doi:10.3390/foods14213672)
Supplement: Supplementary file 1 [file foods-14-03672-s001.zip › foods-3904475-supplementary.pdf]

Table S1. Mean scores (n=292) of sensory consumer desirability of colour, aroma and taste of plant-based beverages as milk alternatives among young consumers with vegan and omnivorous diets.

Sample	Variants of beverages												
	oats	buckwheat	cashews	almonds	soy	pea	rice	coconut	hazelnuts	macadamia	rice-coconut	rice-almond	oat-soy
Women omnivore diet													
Colour	7.27±1.9	6.29±2.2	7.15±2.5	7.85±1.8	7.72±1.8	7.42±2.0	6.56±1.6	8.23±1.6	7.48±1.5	7.90±2.0	7.41±1.6	7.08±2.0	7.22±2.0
Aroma	7.47±1.9	7.64±2.3	8.10±2.6	8.51±1.6	8.23±1.5	7.36±2.0	8.51±1.6	8.64±1.8	8.60±1.6	8.00±1.5	8.29±1.5	7.45±2.0	8.40±2.0
Taste	8.40±1.5	7.35±1.9	7.81±2.0	5.93±1.9	4.60±2.4	7.89±1.8	7.75±1.6	8.52±1.5	8.12±1.5	8.36±1.5	8.10±1.5	7.08±2.0	6.25±1.9
Overal	8.40±1.0	7.35±2.0	7.81±2.0	5.92±1.8	4.63±2.2	7.83±1.6	7.73±2.0	8.51±1.5	8.13±1.7	8.36±1.6	8.07±1.6	7.08±1.9	6.26±1.8
vegan diet													
Colour	7.34±1.9	6.36±1.5	7.05±2.0	7.75±2.0	7.63±2.2	7.18±2.0	6.57±2.1	8.18±1.6	7.43±2.1	7.81±1.2	7.25±2.1	6.97±2.1	7.32±1.5
Aroma	7.64±2.0	7.80±1.2	8.10±2.1	8.60±2.5	8.25±2.1	7.29±2.0	8.55±2.2	8.73±1.5	8.68±2.2	8.11±2.1	8.49±2.1	7.43±2.0	8.41±1.6
Taste	8.47±2.2	7.29±1.8	7.87±1.0	6.28±2.0	4.62±2.0	7.80±1.9	7.67±2.5	8.46±2.2	8.14±2.1	8.35±1.6	8.16±1.5	6.96±1.5	6.09±1.4
Overal	8.44±1.6	7.29±1.8	7.87±1.0	6.29±2.0	4.64±2.0	7.75±2.0	7.64±1.5	8.49±2.1	8.14±1.2	8.35±2.0	8.12±1.5	6.96±2.0	6.11±1.5
Men omnivore diet													
Colour	7.50±1.2	6.33±2.1	6.76±1.6	7.72±1.5	7.50±1.5	7.02±1.0	6.63±1.6	7.92±2.0	7.14±2.2	7.64±1.6	6.88±2.1	6.61±1.0	7.47±1.1
Aroma	7.60±1.1	7.86±2.0	7.85±1.4	8.62±1.6	8.23±1.5	7.03±1.1	8.62±1.6	8.77±2.0	8.84±1.5	7.95±1.7	8.52±1.5	7.25±1.0	8.43±1.2
Taste	8.55±1.5	6.97±2.0	7.87±2.5	6.79±1.6	4.65±2.2	7.50±2.5	7.56±2.1	8.50±2.2	8.03±2.0	8.39±2.0	8.13±1.5	6.32±1.5	5.77±1.5
Overal	8.55±1.4	6.97±2.0	7.87±2.5	6.85±1.6	4.65±2.2	7.49±2.5	7.52±2.2	8.56±2.2	8.05±2.1	8.39±2.0	8.07±1.5	6.32±1.5	5.82±1.5
vegan diet ±													
Colour	7.49±1.5	6.56±1.2	6.96±1.4	7.61±1.5	7.52±1.2	7.15±1.1	6.57±1.2	8.05±1.5	7.45±1.5	7.74±1.7	7.14±1.7	6.85±2.0	7.52±1.6
Aroma	7.49±1.6	6.56±1.5	6.96±1.5	7.61±1.6	7.52±1.3	7.15±1.2	6.57±1.3	8.05±1.1	7.45±1.6	7.74±1.8	7.14±1.5	6.85±2.0	7.52±1.6
Taste	7.83±1.6	7.92±1.6	8.04±1.4	8.61±1.6	8.29±1.4	7.30±1.1	8.63±1.2	8.84±1.1	8.88±1.5	8.15±1.2	8.51±1.6	7.56±2.0	8.48±1.8
Overal	8.51±1.6	7.31±1.6	7.96±1.2	7.01±1.5	4.67±1.4	7.73±1.2	7.59±1.1	8.40±1.2	8.25±1.6	8.41±1.1	8.33±1.5	6.92±1.8	5.78±2.0

Table S2. Mean scores (n=8) of sensory taste profiling of plant-based beverages as milk alternatives.

Sample	Variants of beverages												
	oats	buckwheat	cashews	almonds	soy	pea	rice	coconut	hazelnuts	macadamia	rice-coconut	rice-almond	oat-soy
descriptors of taste													
sweet	2.1±0.3	1.7±0.2	2.8±0.4	1.8±0.3	1.8±0.3	2.1±0.3	1.7±0.3	2.1±0.3	1.5±0.3	2.0±0.3	1.9±0.4	1.5±0.4	1.9±0.4
fatty	1.8±0.2	1.7±0.3	1.8±0.3	1.7±0.3	1.9±0.3	1.9±0.4	0.5±0.1	3.5±0.4	1.5±0.3	3.2±0.4	1.6±0.2	1.2±0.3	1.8±0.3
bitter	1.1±0.3	1.9±0.3	0.6±0.2	1.1±0.2	0.5±0.1	0.6±0.1	0.6±0.2	0.5±0.1	0.7±0.2	0.4±0.1	0.3±0.1	0.8±0.0	0.7±0.2
salty	0.8±0.2	0.7±0.2	0.8±0.2	0.5±0.2	0.9±0.2	0.7±0.2	0.5±0.1	1.3±0.2	0.9±0.3	0.8±0.2	0.7±0.1	0.6±0.2	0.7±0.1
astringent	0.0±0.0	0.2±0.1	0.1±0.1	0.3±0.1	0.8±0.2	0.1±0.1	0.3±0.1	0.0±0.0	0.2±0.1	0.0±0.0	0.0±0.0	0.0±0.0	0.1±0.1
metallic	0.2±0.1	0.3±0.1	0.2±0.1	0.2±0.1	0.3±0.2	0.1±0.1	0.5±0.2	0.6±0.2	0.3±0.2	0.0±0.0	0.0±0.0	0.2±0.1	0.3±0.1
cereal	2.2±0.3	1.8±0.3	0.5±0.2	0.4±0.2	0.2±0.1	0.1±0.1	0.9±0.2	0.2±0.1	0.4±0.2	0.3±0.1	0.4±0.1	0.3±0.1	1.1±0.4
nutty	0.2±0.1	0.2±0.1	1.6±0.3	1.7±0.2	0.4±0.1	0.7±0.2	0.0±0.0	0.7±0.2	1.5±0.3	1.5±0.3	0.5±0.1	0.6±0.2	0.4±0.1
legume	0.0±0.0	0.3±0.1	0.5±0.2	0.4±0.1	2.9±0.3	1.0±0.2	0.2±0.1	0.2±0.1	0.4±0.1	0.3±0.1	0.1±0.1	0.2±0.1	0.9±0.2
watery	0.3±0.1	0.2±0.1	0.2±0.1	0.2±0.1	0.3±0.1	0.2±0.1	1.9±0.3	0.1±0.1	0.3±0.2	0.0±0.0	0.5±0.1	1.2±0.4	0.5±0.1
milky	1.3±0.3	1.0±0.2	1.4±0.2	1.3±0.2	1.2±0.2	1.4±0.2	1.0±0.2	3.2±0.3	1.0±0.2	3.5±0.3	1.7±0.3	1.1±0.3	1.0±0.3
hay-like	0.2±0.1	0.2±0.1	0.0±0.0	0.1±0.1	0.4±0.2	0.0±0.0	0.0±0.0	0.0±0.0	0.2±0.1	0.0±0.0	0.0±0.0	0.1±0.1	0.2±0.1
coconut	0.0±0.0	0.0±0.0	0.0±0.0	0.0±0.0	0.0±0.0	0.0±0.0	0.0±0.0	3.5±0.3	0.3±0.1	0.9±0.3	1.2±0.3	0.0±0.0	0.2±0.1

S.1. Survey questionnaire template.

Please mark your answer with an "X" in the box.

Participation in the survey is voluntary. By completing the survey, you consent to participate in the study. You may withdraw from the study at any time.

1. Gender
☐ Female ☐ Male

2. Age
☐ Under 18
☐ 18 – 24
☐ 25 – 30
☐ Over 30

3. What diet do you follow?
☐ Vegan diet
☐ Traditional diet
☐ Other diet, what kind?

4. Why do you drink plant-based drinks? (You can select more than one answer)

☐ I am on a vegan diet
☐ I am lactose intolerant/allergic to dairy
☐ Due to the positive sensory characteristics of plant-based drinks
☐ I don't like cow's milk
☐ I am on a diet that reduces saturated fat intake
☐ Other diet, what kind?

4. Which plant-based drink do you prefer most?

☐ Almond
☐ Soy
☐ Coconut
☐ Oat
☐ Buckwheat
☐ Pea
☐ Rice-Coconut
☐ Oat-Soy

☐ Rice
☐ Other, specify…

7. How often do you drink plant-based drinks?

☐ Several times a day
☐ Once a day
☐ Several times a week
☐ Once a week
☐ Several times a month
☐ Several times a year;
☐ Less than ten times/never

8. Please rank the factors influencing your choice of plant-based drink (from most important to least important):

☐ Price
☐ Taste
☐ Packaging
☐ Opinions of friends
☐ Ingredients
☐ Other, specify
